# The Reemergence of Seasonal Respiratory Viruses in Houston, Texas, after Relaxing COVID-19 Restrictions

**DOI:** 10.1128/Spectrum.00430-21

**Published:** 2021-09-08

**Authors:** Parsa Hodjat, Paul A. Christensen, Sishir Subedi, David W. Bernard, Randall J. Olsen, S. Wesley Long

**Affiliations:** a Center for Molecular and Translational Human Infectious Diseases Research, Department of Pathology and Genomic Medicine, Houston Methodist Research Institute and Houston Methodist Hospital, Houston, Texas, USA; Johns Hopkins Hospital

**Keywords:** COVID-19, coronavirus, influenza, masking, parainfluenza virus, respiratory syncytial virus

## Abstract

Measures intended to limit the spread of the severe acute respiratory syndrome coronavirus 2 (SARS-CoV-2) virus at the start of the coronavirus disease 2019 (COVID-19) pandemic resulted in a rapid decrease in other respiratory pathogens. Herein, we describe the trends of respiratory pathogens in a major metropolitan health care system central microbiology reference laboratory before and during the COVID-19 pandemic, with attention to when COVID-19 mitigation measures were implemented and relaxed. During the initial lockdown period, COVID-19 was the primary respiratory pathogen detected by multiplex respiratory panels. As COVID-19 containment measures were relaxed, the first non-COVID respiratory viruses to return to prepandemic levels were members of the rhinovirus/enterovirus family. After the complete removal of COVID-19 precautions at the state level, including an end to mask mandates, we observed the robust return of seasonal coronaviruses, parainfluenza virus, and respiratory syncytial virus. Inasmuch as COVID-19 has dominated the landscape of respiratory infections since early 2020, it is important for clinicians to recognize that the return of non-COVID respiratory pathogens may be rapid and significant when COVID-19 containment measures are removed.

**IMPORTANCE** We describe the return of non-COVID respiratory viruses after the removal of COVID-19 mitigation measures. It is important for the public and physicians to recognize that, after months of COVID-19 being the primary driver of respiratory infection, more typical seasonal respiratory illnesses have returned, and this return is out of the normal season for some of these pathogens. Thus, clinicians and the public must now consider both COVID-19 and other respiratory illnesses when a patient presents with symptomatic respiratory illness.

## OBSERVATION

Implementation of measures to limit severe acute respiratory syndrome coronavirus 2 (SARS-CoV-2) transmission during the coronavirus disease 2019 (COVID-19) pandemic coincided with a marked decrease in infections caused by other respiratory viruses (1). The decrease was due, in part, to masking, social distancing, closure of schools and businesses, and other efforts targeted at limiting disease spread (2).

Houston Methodist Hospital System is a large multihospital system in one of the most diverse cities in the United States. The centralized reference microbiology laboratory performs a variety of testing, including the BioFire respiratory pathogen panel, which tests for 22 different pathogen targets. Our testing methods for COVID-19, including genome-sequencing efforts, have been published previously (3–5).

**FIG 1:**
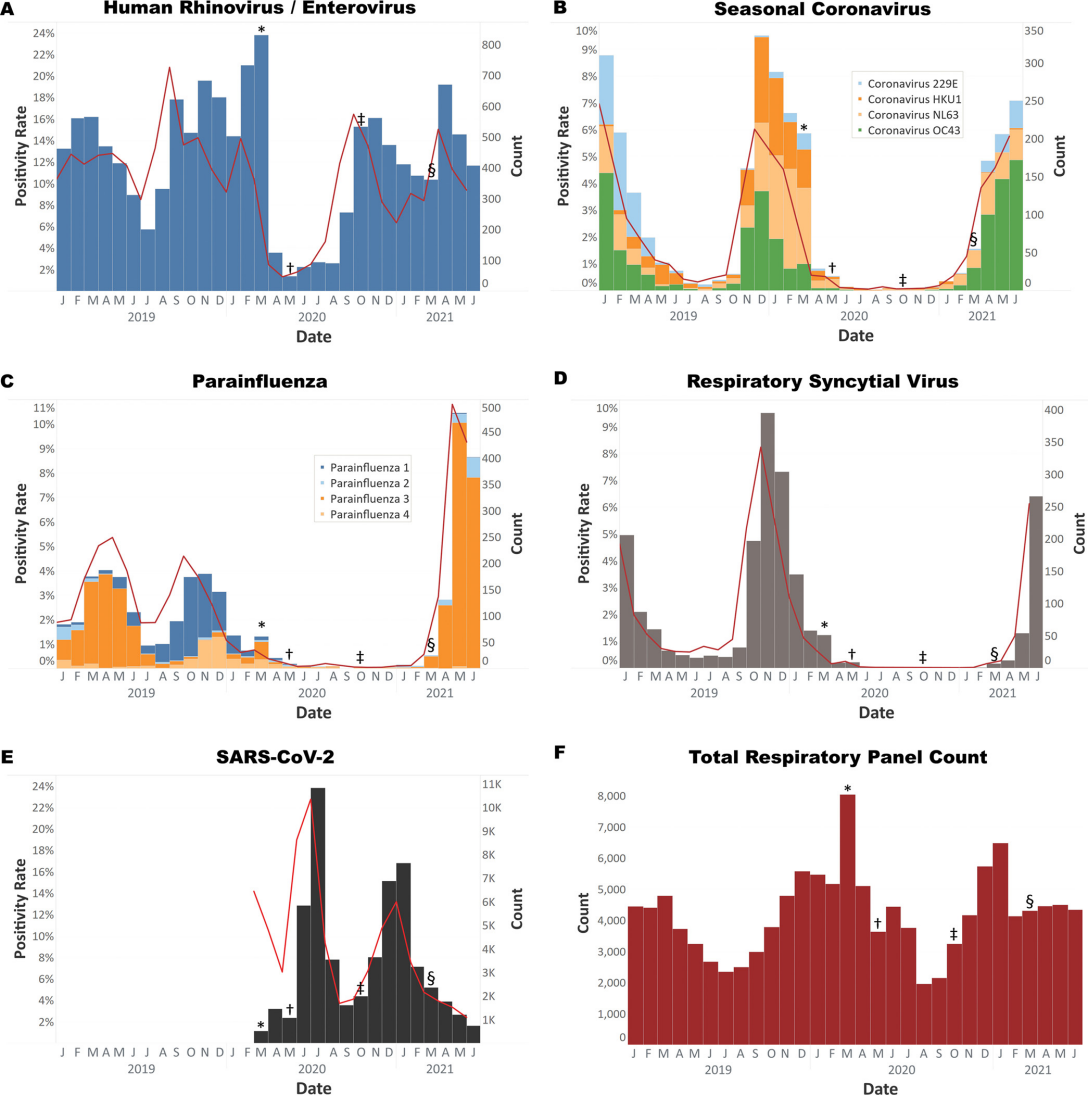
Histograms of respiratory virus tests from 1 January 2019 to 25 May 2021. (A) Rhinovirus/enterovirus was the first virus to rebound to prepandemic levels. (B) Coronaviruses 229E, HKU1, NL63, and OC43. OC43 has been prevalent since March 2021. (C) Parainfluenza viruses 1 to 4. Parainfluenza 3 has been prevalent since March 2021. (D) Respiratory syncytial virus has also increased in prevalence since March 2021. (E) SARS-CoV-2 testing volume and positivity from March 2020 through June 2021. (F) Respiratory panel test volume from January 2020 through June 2021. Symbols indicate the implementation and relaxation of COVID-19 precautions. *, March 2020, start of pandemic lockdown measures; †, May 2020, phase one reopening of Texas; ‡, October 2020, additional reopening measures; §, March 2021, removal of all COVID restrictions, including elimination of mask mandates. All tests were performed on the Biofire respiratory pathogen panel, with the exception of (E) which represents all SARS-CoV-2 PCR testing performed using multiple test vendors.

Early in the public health response to COVID-19 in Houston, the Houston Livestock Show and Rodeo was cancelled on 11 March 2020 and a citywide stay-at-home order was implemented on 25 March 2020 (https://abc13.com/houston-rodeo-coronavirus-update-texas-cancellation-livestock-show-and/6003475/ and https://www.houstontx.gov/mayor/press/2020/stay-home-work-safe-order.html, last accessed 25 May 2021). Subsequently, rates of influenza virus, respiratory syncytial virus (RSV), rhinovirus/enterovirus, and seasonal coronavirus infections, as diagnosed by the respiratory pathogen panel in the Houston Methodist Hospital centralized microbiology laboratory, declined rapidly (Fig. 1) (https://flu.houstonmethodist.org, last accessed 25 May 2021). Similar declines can be observed both in influenza surveillance data and respiratory pathogen panel data from the United States (https://www.cdc.gov/flu/weekly/index.htm and https://syndromictrends.com, last accessed 1 July 2021) and other countries (6, 7).

As COVID-19 measures were gradually relaxed starting in May 2020, very low levels of non-SARS-CoV-2 respiratory pathogens were detected even as cases of COVID-19 began to rise (https://gov.texas.gov/uploads/files/press/EO-GA-23_phase_two_expanding_opening_COVID-19.pdf, last accessed 25 May 2021) (8). Levels of non-COVID respiratory infections remained exceedingly low through the summer (10 to 28 rhinovirus/enterovirus cases per week [cpw], 0 to 4 cpw of influenza A and influenza B virus, and 0 to 1 cpw of RSV). In September 2020, rhinovirus/enterovirus cases began increasing to prepandemic levels as schools reopened and many remaining measures were relaxed in October 2020 (97 to 156 rhinovirus/enterovirus cases per week, October through December 2020) (Fig. 1A) (https://gov.texas.gov/uploads/files/press/EO-GA-32_continued_response_to_COVID-19_IMAGE_10-07-2020.pdf, last accessed 25 May 2021).

The first week of March 2021, the Texas governor announced that the remaining measures were being eliminated and face masks could no longer be mandated by state or local government (https://open.texas.gov/uploads/files/organization/opentexas/EO-GA-34-opening-Texas-response-to-COVID-disaster-IMAGE-03-02-2021.pdf, last accessed 25 May 2021). That same month, we began to observe a marked increase in rhinovirus/enterovirus, parainfluenza virus, and seasonal coronavirus infections (Fig. 1B to D). In Houston, seasonal coronaviruses typically peak during the winter months, with very low levels observed during the summer. We are now observing a month-over-month increase in parainfluenza and seasonal coronaviruses which would be considered out of season compared to their typical seasonality. Out-of-season increases in RSV have been reported elsewhere when COVID-19 measures were relaxed (https://www.abc.net.au/news/2021-02-24/rsv-cases-surging-in-south-east-queensland/13186788, last accessed 25 May 2021). We have also seen a recent increase in RSV cases in May 2021 (0 to 1 cpw between 1 June 2020 and 1 March 2021, now increased to 66 cpw in June 2021), although our patient population is primarily comprised of adults. Rhinovirus/enterovirus cases have also increased from 82 to 106 cpw in January and February 2021 to 143 to 156 cpw from the end of March through April 2021, decreasing to just over 100 cpw in June 2021.

Inasmuch as the incremental relaxation of COVID-19 prevention measures affected respiratory infection rates over time, the recent discontinuation of mask mandates in March 2021 has coincided with a rapid increase in a variety of non-COVID respiratory pathogens. These observations are important for clinicians to consider as they evaluate patients with respiratory infections in the coming months. They also underscore the high effectiveness of nonpharmacologic preventative measures like masking and social distancing in preventing the spread of respiratory pathogens.
